# Assessing cervical intervertebral disc height on MRI and X-ray versus CT; a single center retrospective cohort study

**DOI:** 10.1016/j.bas.2026.105927

**Published:** 2026-01-08

**Authors:** Esther van Santbrink, Toon Boselie, Valérie Schuermans, Nykle Krijgsveld, Rob de Bie, Anouk Smeets, Henk van Santbrink

**Affiliations:** a Dept. of Neurosurgery, Maastricht University Medical Centre, Maastricht, the Netherlands; b Dept. of Neurosurgery, Zuyderland Medical Centre, Heerlen, the Netherlands; c CAPHRI Institute for Public Health and Primary Care, Maastricht University, Maastricht, the Netherlands; d Faculty of Health, Medicine, and Life Sciences, Maastricht University, Maastricht, the Netherlands; e Department of Epidemiology, Maastricht University, Maastricht, the Netherlands

**Keywords:** Cervical intervertebral disc height, MRI, X-ray, CT, Reliability and agreement

## Abstract

**Introduction:**

Cervical intervertebral disc height is often used to score cervical degenerative disc disease (CDDD) and can also aid in pre-operative surgical planning. For this purpose, MRI and X-ray imaging are routinely used. However, the reliability and agreement with computed tomography (CT), the gold standard for linear measurements, remain unknown.

**Research question:**

To assess the reliability and agreement of intervertebral disc height measurements between MRI, X-ray, and CT.

**Material and methods:**

Data was collected from all patients that received an MRI, X-ray, and CT within a timeframe of 6 months at Zuyderland Medical Center (ZMC) between 2014 and 2024. Mid, anterior, and posterior cervical intervertebral disc heights were measured from C2-C3 to C6-C7 in all three imaging modalities. 120 patients were included. The intraclass correlation coefficient (ICC) was calculated for the inter- and intraobserver reliability. A linear mixed model with Bonferroni correction was used to compare outcomes.

**Results:**

ICCs ranged from moderate to excellent for X-ray. ICCs ranged from good to excellent for MRI and CT after sensitivity analysis for equal slice selection. Mean difference in disc height was .1 mm (p = 0.163) between MRI and X-ray, 1.3 mm (p < 0.001) between MRI and CT, and 1.3 mm (p < 0.001) between X-ray and CT.

**Discussion and conclusion:**

MRI and X-ray exhibit a systematic bias in the measurement of cervical intervertebral disc height when compared to CT. CT should be considered for the assessment of disc height. The mid-disc measurement is recommended to ensure high reliability.

## Highlights

• MRI and X-ray exhibit a systematic bias in the measurement of cervical intervertebral disc height compared to CT.
• If precise disc height assessment is critical, CT imaging should be considered.
• The mid-disc measurement is recommended to ensure high reliability.

## 1 Introduction

Cervical degenerative disc disease (CDDD) is present in 54 % of the general population and incidence increases with age (Tao et al., 2021). Several grading systems for CDDD are described, measuring the presence of sclerosis and osteophytes, and the cervical intervertebral disc height (Kellgren and Lawrence, 1957; Gore et al., 1986; Walraevens et al., 2009; Lawrence, 1969).

Evaluation of these degenerative changes can aid in preoperative decision-making (Nouri et al., 2017; Kato et al., 2018). The choice for an implant in an anterior surgical approach may depend on the preoperative intervertebral disc height. Severe loss of disc height, defined as remaining disc height less than 3 mm (mm), is for example a contraindication for the implantation of a disc prosthesis (Nunley et al., 2020; Patel et al., 2024). Overcompensation of intervertebral disc height is associated with a decrease in range of motion (ROM) (Wang et al., 2021). Overcompensation of disc height in anterior cervical discectomy and fusion (ACDF), defined as a postoperative increase of 2 mm or more after cage placement, is associated with an increased risk of cage subsidence and the development of adjacent segment disease (Hsueh et al., 2023; Duey et al., 2024). Choosing the correct implant and implant size in anterior cervical surgery is essential for a good outcome.

For the lumbar spine, different techniques to measure intervertebral disc height on radiographs (X-rays), magnetic resonance imaging (MRI), and computed tomography (CT) have been described and validated (Hurxthal, 1968; Dabbs and Dabbs, 1990; Frobin et al., 1997; Fyllos et al., 2018; Boos et al., 1996; Abdollah et al., 2019; Spahn et al., 2024). Normative values for lumbar intervertebral disc height on computed tomography (CT) have been reported (Bach et al., 2018).

For the cervical spine, CT derived normative values are also available (Shin et al., 2024). CT is used to assess bone structures as a complementary imaging modality and can accurately assess degenerative changes in the cervical spine (Rydman et al., 2019). However, it is not routinely performed for degenerative neurosurgical diagnoses and the comparability of disc height measurements with measurements obtained from other imaging modalities has not yet been established. Conventional X-rays are commonly used to assess cervical intervertebral disc height (Walraevens et al., 2009). Hurxthal's method, measuring the mid-vertebral disc height on a lateral X-ray, is often used due to its user-friendly nature (Hurxthal, 1968; van Eerd et al., 2021). X-ray has several advantages compared to other imaging modalities, such as wide availability, low costs and acquisition in a weight-bearing position. However, neurological symptoms originating from the cervical spine are primarily evaluated on MRI. If compression of neurogenic structures is present, a surgical procedure may be recommended without the need for additional X-ray imaging. MRI has been proposed for evaluating degenerative changes in the cervical spine, and a strong association between MRI and X-ray findings in assessing these changes has been reported (Nouri et al., 2016; Moll et al., 2018; Kolstad et al., 2005).

The primary aim of this study is to investigate the reliability and agreement of cervical intervertebral disc height measurements between MRI, X-ray, and CT.

## 2 Methods

A single-center retrospective cohort study was performed at Zuyderland Medical Center (ZMC), the Netherlands, a tertiary spine center. This study was approved by the Medical Ethical Review Committee of Zuyderland MC (METCZ20240088). This research was conducted in accordance with the Declaration of Helsinki (2013). As this retrospective study does not fall under the Dutch Medical Research Involving Human Subjects Act (WMO), the ethics committee waived the requirement for informed consent.

A consecutive series of patients aged over 18, who received a neurologically related diagnosis code between January 2014 and June 2024, and received an MRI, X-ray, and CT of the cervical spine within a timeframe of 6 months at ZMC were included.

### 2.1 Radiological assessment

Cervical intervertebral disc height of levels C2-C3 to C6-C7 were analyzed on MRI, X-ray, and CT. If the intervertebral disc of a patient was affected by trauma, congenital deformities, inflammatory diseases (e.g., Ankylosing spondylitis, Forestier's disease), or surgical procedures, the affected disc was excluded. If a level had been operated in one or two of the imaging modalities, it was excluded from analysis for all three modalities.

**Fig. 1:**
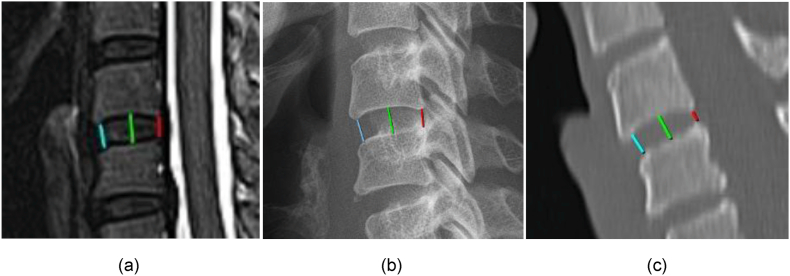
Example of C4-C5 mid disc height (green line), anterior disc height (blue line), and posterior disc height (red line) measured in the same patient; (a) MRI; (b) X-ray; (c) CT.

The following three locations were assessed per level: mid vertebral disc height (green line), anterior vertebral disc height (blue line), and posterior vertebral disc height (red line) (Fig. 1).

CT measurements were performed using the “bone” setting. MRI measurements were performed on T2-weighted images, TSE (turbo spin echo). For both CT and MRI, the mid-sagittal plane was independently selected and checked with a localizer on the transverse plane by two observers (EvS, NK). The sagittal slice number was noted. Slice thickness was set at 3 mm for both CT and MRI.

The two observers performed all measurements and were blinded for each other's measurements to determine interobserver reliability. One observer (NK) repeated the measurements of 20 randomly chosen subjects to determine the intraobserver reliability, with an interval of 4 weeks (Bujang and Baharum, 2017).

### 2.2 Sample size calculation

The sample size calculation was based on two observers achieving an intraclass correlation coefficient (ICC) of .792 for cervical disc height measured on CT (Shin et al., 2024). To achieve an ICC of .792 with a precision of .1 and a 95 % confidence interval (CI), the study required a sample size of 55 patients per level (Arifin, 2018).

### 2.3 Main study endpoints

The primary outcome was the overall difference in cervical intervertebral disc height measured on MRI, X-ray, and CT. Disc height was assessed from levels C2-C3 to C6-C7. For the measurement of the cervical intervertebral disc height on MRI and CT, the mid-sagittal plane was used, for X ray a lateral image was used.

Secondary outcomes included the differences between levels C2-C3 to C6-C7 and between the anterior, mid, and posterior measurement location.

**Fig. A1:**
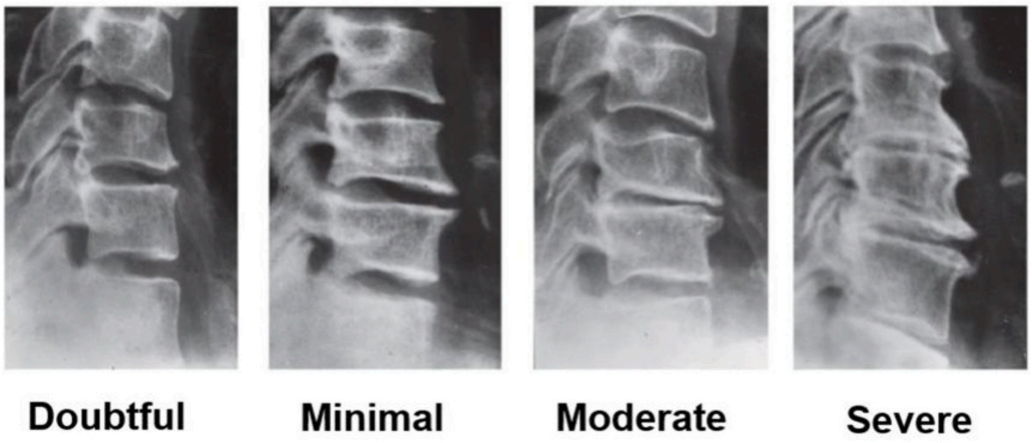
Kellgren-Lawrence score.

The Kellgren-Lawrence (KL) score was used to assess degenerative changes on lateral cervical X-rays (Figure A1, Table A1, Appendix 1). The aim was to evaluate whether the degree of degeneration influenced the measurement reliability. The score ranges from 0 to 4, with higher scores indicating more severe degeneration at each cervical level.

Age, sex, and image date were retrieved from electronic patient records.

### 2.4 Data analysis

Patient data and clinical characteristics were presented as means and standard deviation (±SD) for continuous variables and absolutes and percentages (%) for categorical variables.

ICCs were calculated to determine interobserver and intraobserver reliability for the three locations of all included levels. A two-way mixed effects model, testing for absolute agreement, was used. Values less than .4 were rated as poor reliability, values between .4 and .6 were rated as moderate reliability, values between .6 and .8 were rated as good reliability, and values greater than .8 were rated as excellent reliability (Landis and Koch, 1977). A weighted Cohen's kappa was calculated for the assessment of the Kellgren-Lawrence score.

A sensitivity analysis was performed to analyze the influence of differences in slice selection on inter- and intraobserver reliability scores for MRI and CT.

A linear mixed model (LMM) with restricted maximum likelihood was used to assess differences in cervical intervertebral disc height across sex, age, the three imaging modalities, levels C2-C3 to C6-C7, and measurement locations. Patient ID was included as a random intercept to account for the 45 repeated measures within each subject. Sex, age, imaging modality, level, and location were fixed factors, and their interactions were entered as fixed effects. A subgroup factor indicating whether the time between imaging was ≤30 days or >30 days, was added as an additional fixed effect. Estimated marginal means and pairwise comparisons were conducted using an LMM with a Bonferroni adjustment. Disc height was the dependent variable.

The dataset of one observer was used for LMM. All statistical analyses were performed using IBM SPSS Statistics Version 28 (IBM SPSS Statistics for Windows, 2021).

### 2.5 Data selection

In total 830 patients were retrieved from the electronic patient files that received an MRI of the cervical spine for neurological diagnostic purposes. Among them, 141 patients also received a cervical X-ray and CT within a timeframe of six months. Seven patients had two sets of the three imaging modalities of which the first complete set was used.

After verification, 25 sets were excluded for the following reasons: fusion of all vertebrae (n = 7), inadequate imaging/missing images/incorrectly labeled (n = 4), and diagnosis of ankylosing spondylitis (n = 3).

**Fig. 2:**
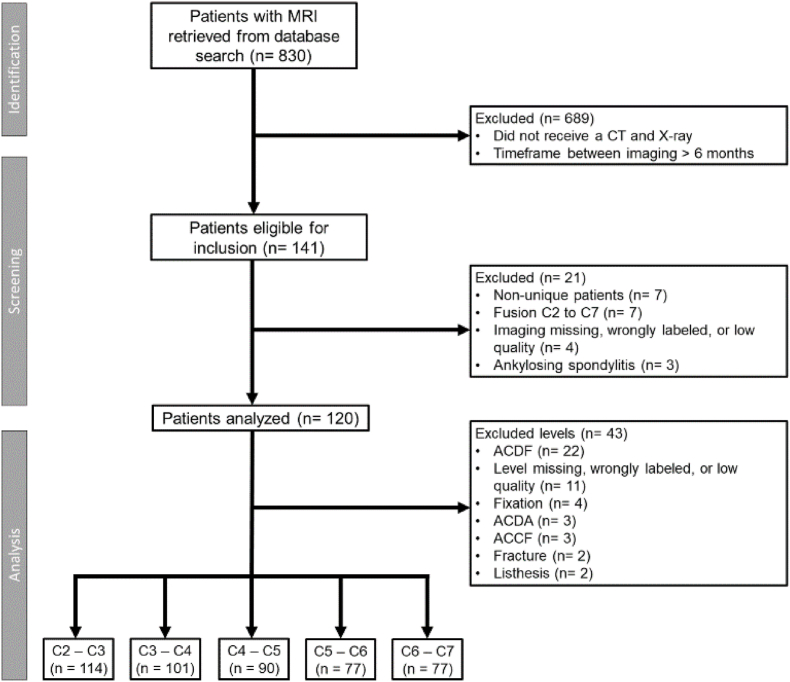
Flowchart of participant selection process and number of individual levels analyzed.

In total 120 patients were available for analysis. After excluding specific vertebral levels affected by fusion, fractures, spondylolisthesis, previous discectomy or corpectomy, or inadequate/missing imaging across all three modalities, the final number of levels available for analysis was 114 for C2-C3, 101 for C3-C4, 90 for C4-C5, 77 for C5-C6, and 77 for C6-C7. Fig. 2 shows a flowchart of the selection process.

## 3 Results

Mean age was 68 years (SD ± 12), and 55 % of patients were male. Mean time between first and last imaging was 49 days (SD ± 47), with a median of 33 days (range 0–174), as depicted in Table 1.

**Table 1:** Patient and clinical characteristics at baseline.

Characteristic	n = 120
Gender (%)	
- Male	66 (55)
- Female	54 (45)
Age (years);	
Mean ± SD	68 ± 12
Median (range)	68 (39–94)
Time between imaging (days);	
Mean ± SD	49 ± 47
Median (range)	33 (0–174)

Mean disc height for each modality, level, and measurement location is presented in Table 2. For X-ray, the mean ICC ranged from .532 to .963 for intraobserver reliability and from .552 to .885 for interobserver reliability. After sensitivity analysis for equal slice selection, the mean ICC for intraobserver reliability ranged from .668 to .983 for MRI and from .639 to .993 for CT. Mean ICC for interobserver reliability ranged from .806 to .949 for MRI and from .768 to .960 for CT (Table 2).

**Table 2:** Mean disc height per modality, level, and location with inter-class correlation for absolute agreement, intra- and interobserver reliability.

		Mean disc height (mm)				Intra observer reliability with equal slices			Inter observer reliability with equal slices		
		MRI (SD)	X-ray (SD)	CT (SD)	n	MRI (95 %CI)	X-ray (95 %CI)	CT (95 %CI)	MRI (95 %CI)	X-ray (95 %CI)	CT (95 %CI)
**C2-C3**	**Mid.** **Ant.** **Post**	5.9 (1.1) 4.2 (.9) 3.9 (1.0)	6.9 (1.4) 4.6 (1.3) 3.2 (1.1)	4.9 (1.1) 3.0 (.8) 2.3 (.8)	113	.898 (.505–.974) .841 (.373–.957) .795 (.432–.936)	.848(.649–.939) .532(.139–.786) .583(.195–.815)	.639 (.247–.855) .695 (.331–.880) .707 (.348–.886)	.949 (.891–.973) .881 (.818–.922) .908 (.861–.940)	.669 (.486–.783) .792 (.713–.852) .552 (.411–.667)	.915 (.839–.951) .859 (.668–.929) .791 (.466–.900)
**C3-C4**	**Mid.** **Ant.** **Post**	5.7 (1.1) 3.9 (1.1) 3.3 (1.0)	6.5 (1.6) 4.3 (1.5) 2.7 (1.2)	4.7 (1.1) 2.6 (.9) 2.1 (.9)	101	.980 (.934–.994) .668 (.202–.891) .882 (.593–.967)	.929(.823–.973) .853(.650–.942) .616(.223–.837)	.949 (.851–.983) .911 (.752–.970) .808 (.516–.933)	.914 (.777–.958) .816 (.724–.879) .865 (.794–.912)	.833 (.588–.917) .842 (.775–.891) .723 (.591–.813)	.960 (.937–.975) .886 (.824–.927) .845 (.520–.933)
**C4-C5**	**Mid.** **Ant.** **Post.**	5.4 (1.1) 4.0 (1.1) 3.3 (1.0)	6.2 (1.4) 4.1 (1.6) 2.6 (1.2)	4.3 (1.1) 2.4 (1.0) 2.0 (.9)	90	.983 (.938–.996) .836 (.493–.956) .905 (.591–.977)	.935(.819–.978) .857(.635–.949) .754(.425–.909)	.993 (.875–.999) .934 (.783–.982) .892 (.665–.969)	.928 (.759–.969) .847 (.761–.904) .889 (.823–.931)	.754 (.484–.869) .869 (.808–.912) .759 (.656–.835)	.905 (.850–.941) .828 (.735–.891) .870 (.774–.923)
**C5-C6**	**Mid.** **Ant.** **Post.**	4.3 (1.3) 3.5 (1.2) 2.5 (.9)	4.9 (1.7) 3.3 (1.5) 1.7 (1.1)	3.4 (1.3) 1.9 (1.1) 1.3 (.8)	77	.940 (.727–.989) .861 (.403–.975) .808 (.054–.967)	.958(.678–.992) .891(.394–.977) .879(.553–.971)	.950 (.683–.993) .920 (.531–.988) .891 (.465–.984)	.933 (.880–.961) .815 (.704–.886) .806 (.512–.909)	.847 (.567–.930) .872 (.805–.916) .623 (.361–.774)	.919 (.864–.952) .869 (.764–.926) .828 (.636–.912)
**C6-C7**	**Mid.** **Ant.** **Post**	4.9 (1.6) 4.0 (1.4) 2.8 (1.1)	5.6 (1.9) 3.8 (1.9) 1.9 (1.5)	3.9 (1.4) 2.3 (1.3) 1.5 (1.4)	77	.982 (.910–.997) .909 (.431–.985) .941 (.726–.989)	.932(.261–.988) .963(.028–.995) .953(.811–.989)	.890 (.581–.974) .831 (.445–.959) .760 (.228–.941)	.912 (.714–.963) .907 (.844–.945) .854 (.626–.932)	.852 (.683–.922) .885 (.825–.925) .690 (.365–.836)	.939 (.898–.964) .897 (.826–.940) .768 (.517–.880)

Reliability is scored as <.4 = Poor, .4–.6 = Moderate, .6–.8 = Good, >.8 = Excellent.

An overview of all ICCs per modality, level, and location with the number of patients based on can be found in Tables A2-A5.

The weighted Cohen's kappa showed poor reliability for the Kellgren-Lawrence score (Table 3). Therefore, a sensitivity analysis based on level of degeneration was not performed.

**Table 3:** Weighted Cohen's Kappa reliability analysis for Kellgren-Lawrence score attribution.

Weighted Cohen's Kappa	Intraobserver (95 % CI)	Interobserver (95 % CI)	n
***KG C2-C3***	.337 (−.041–.715)	.160 (.059–.260)	17/114
***KG C3-C4***	.758 (.550–.967)	.341 (.220–.461)	16/101
***KG C4-C5***	.737 (.516–.958)	.316 (.203–.429)	13/90
***KG C5-C6***	.667 (.379–.954)	.320 (.213–.428)	7/77
***KG C6-C7***	.640 (.396–.884)	.398 (.284–.513)	7/77

Weighted Cohen's Kappa scored as <.4 = Poor, .4–.6 = Moderate, .6–.8 = Good, >.8 = Excellent.

**Fig. 3:**
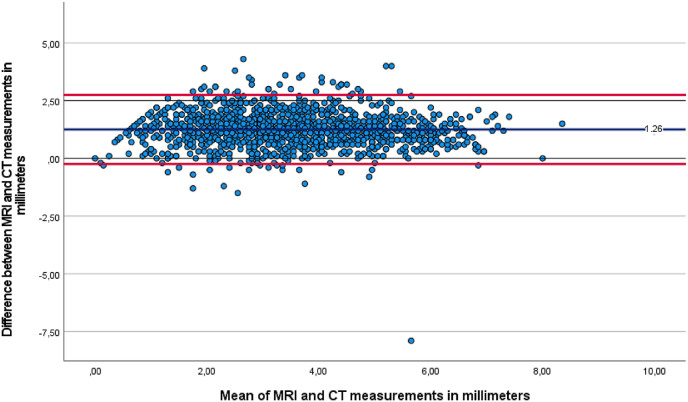
Bland-Altman plot of mean MRI and CT measurement against measured differences between MRI and CT.

**Fig. 4:**
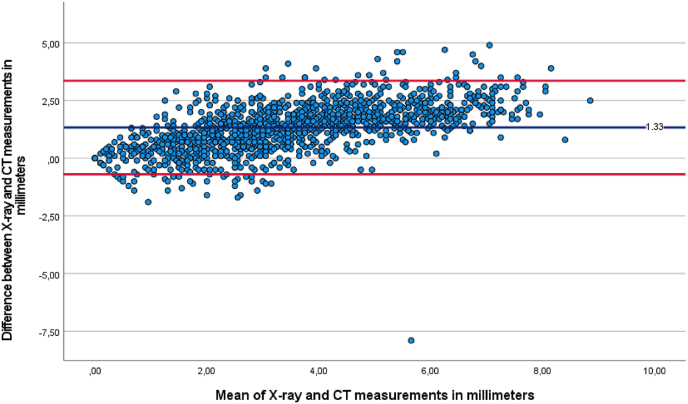
Bland-Altman plot of mean X-ray and CT measurement against measured differences between X-ray and CT.

**Fig. 5:**
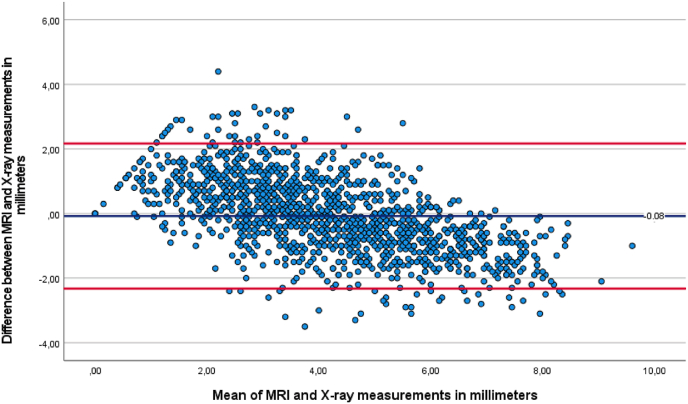
Bland-Altman plot of mean MRI and X-ray measurement against measured differences between MRI and X-ray.

The mean difference was .1 mm (95 % CI: .2 to .0, p = 0.163) between MRI and X-ray, 1.3 mm (95 % CI: 1.2 to 1.4, p < 0.001) between MRI and CT, and 1.3 mm (95 % CI: 1.2 to 1.4, p < 0.001) between X-ray and CT, as seen in Table 4. Bland–Altman plots were generated to illustrate the differences between modalities relative to their mean (Fig. 3, Fig. 4, Fig. 5).

**Table 4:** Pairwise comparisons.

Parameter		Mean difference	p-value^a^	95 CI interval	
				Lower bound	Upper bound
***MRI***	***X***	−.08	.163	−.17	.02
	***CT***	1.26^a^	<.001	1.16	1.35
***X-ray***	***MRI***	.08	.163	−.02	.17
	***CT***	1.33^a^	<.001	1.24	1.43
***CT***	***MRI***	−1.26^a^	<.001	−1.35	−1.16
	***X***	−1.33^a^	<.001	−1.43	−1.24

Based on estimated marginal means.

The mean difference is significant at the,05 level.

Adjustment for multiple comparisons: Bonferroni.

Disc height was .39 mm (95 % CI .141–.635, p = 0.002) higher in males than females. Age was negatively associated with disc height (β = −.0189 per year, 95 % CI −.0288 to −.0091, p < 0.001). Time between imaging (≤30 vs. >30 days) had no significant effect on disc height (.160 mm difference, 95 % CI −.101 to .421, p = 0.227) and the mean difference between MRI and CT and X-ray and CT remained 1.3 mm, the difference between MRI and X-ray was 0 mm.

**Fig. 6:**
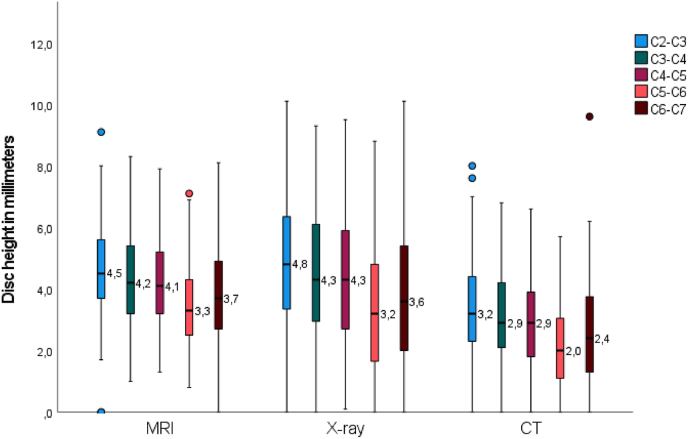
Boxplot of mean disc height of raw data per modality and level.

**Fig. 7:**
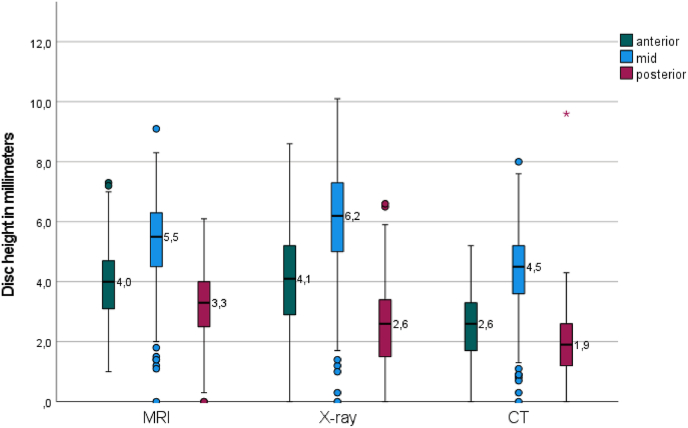
Boxplot of mean disc height of raw data per modality and location.

Compared to C6–C7, disc height was .80 mm (p < 0.001) higher at C2–C3, .46 mm (p < 0.001) higher at C3–C4, and .31 mm (p < 0.001) higher at C4–C5, while C5–C6 showed a .35 mm (p < 0.001) lower disc height. Relative to the posterior location, the mid location showed 2.69 mm (p < 0.001) greater disc height, and the anterior location showed a .95 mm (p < 0.001) greater disc height. Fig. 6 illustrates the mean difference between the modalities and levels of the raw data in a boxplot. Fig. 7 illustrates the mean difference between the modalities and location of the raw data in a boxplot.

## 4 Discussion

This study investigated the reliability and measurement agreement of cervical intervertebral disc height on MRI and X-ray compared to CT. The findings demonstrate that disc height values derived from MRI and X-ray differ significantly from those obtained from CT, indicating a lack of agreement between imaging modalities. Disc height measurements showed high reliability within each modality. Based on these results, CT should be used for the assessment of disc height and the mid-disc measurement is recommended to ensure high reliability.

**Fig. A2:**
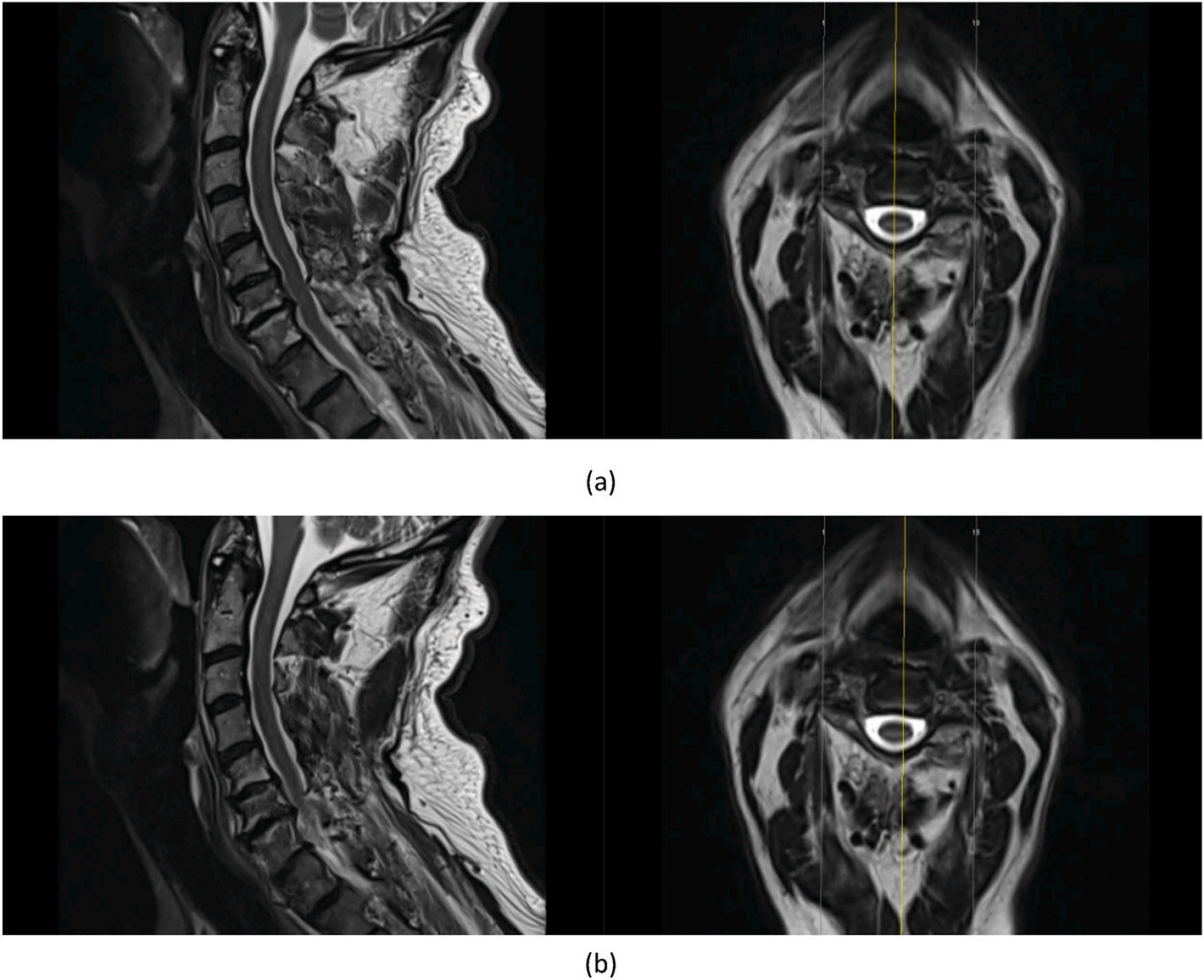
Example of difference in slice selection on sagittal and transverse MRI, with localizer (yellow line) present on the transverse plane; (a) Slice 9; (b) Slice 10.

**Fig. A3:**
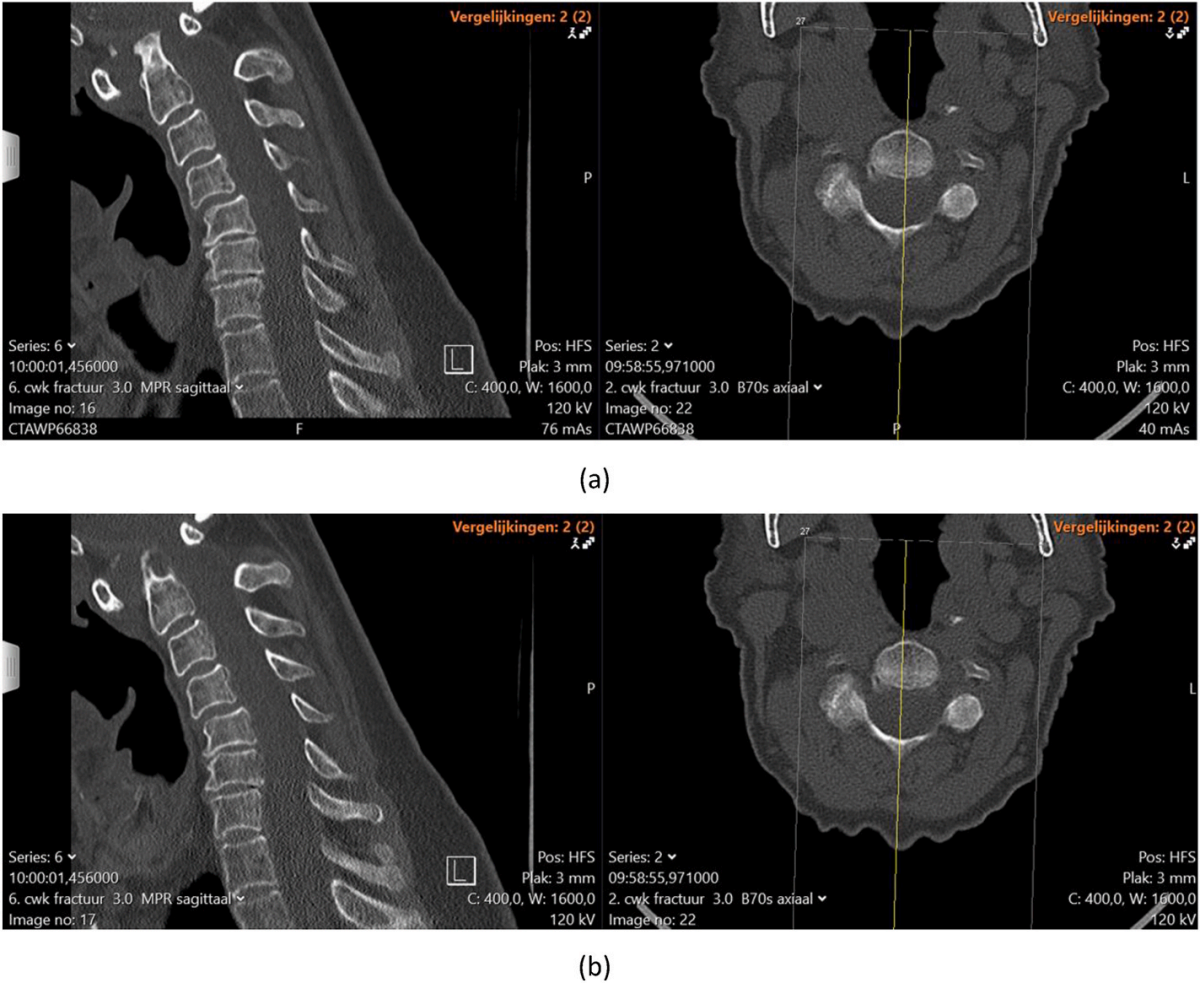
Example of difference in slice selection on sagittal and transverse CT, with localizer (yellow line) present on the transverse plane; (a) Slice 16; (b) Slice 17.

Overall, the mid-disc height measurement, also known as Hurxthal's method, resulted in the highest ICC value. Abdollah et al. compared the mid-disc measurement to the average of the anterior and posterior disc height, also known as Dabb's method, of level L4-L5 and L5-S1 on MRI (Abdollah et al., 2019). Inter- and intraobserver reliability demonstrated excellent agreement for both levels and methods. In this study, the observers were presented with the whole MRI and CT series and were blinded to the mid-sagittal slice selection of the other observer as in the real world. To illustrate the difficulty of defining the true mid-sagittal slice, MRI and CT examples are shown in Figures A2 and A3. Sensitivity analysis for equal slice selection resulted in excellent agreement for both MRI and CT comparable to Abdollah et al. (2019). If disc height is assessed in daily practice, slice selection should be carefully documented, otherwise reliability decreases substantially. ICC values may also be influenced by disc height, with lumbar discs being generally higher than cervical discs and therefore less affected by measurement error, as well as by imaging technique such as the choice of MRI sequence.

In the assessment of interobserver reliability on X-ray, the anterior measurement often exhibited higher reliability than the mid measurement. Spahn et al. compared the reliability of an X-ray to an MRI for three methods: Hurxthal, Dabb, and Fyllos (Spahn et al., 2024). Fyllos' method is the average of the anterior, mid, and posterior disc height. The interobserver reliability for Dabb's and Fyllos' method was higher on X-ray compared to MRI. In contrast, the ICC for Hurxthal's method is higher for MRI compared to X-ray. This can be attributed to differences in imaging techniques. X-ray is a two-dimensional imaging technique, possibly leading to overprojection of the endplates. Overprojection makes it more difficult to determine the disc's apex of the concavity, thereby interfering with the mid measurement, Hurxthal's method.

**Fig. A4:**
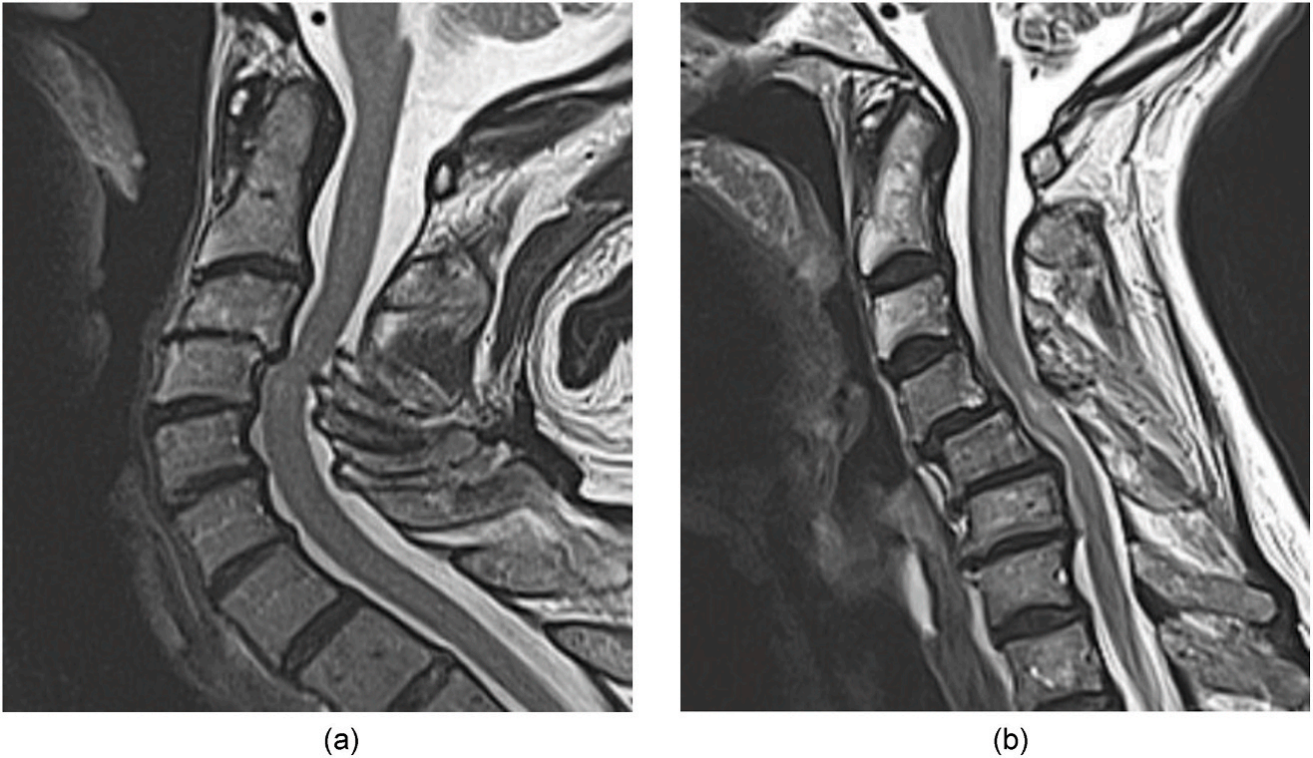
Example of severe degeneration of the cervical spine on MRI; (a) Example 1; (b) Example 2.

**Fig. A5:**
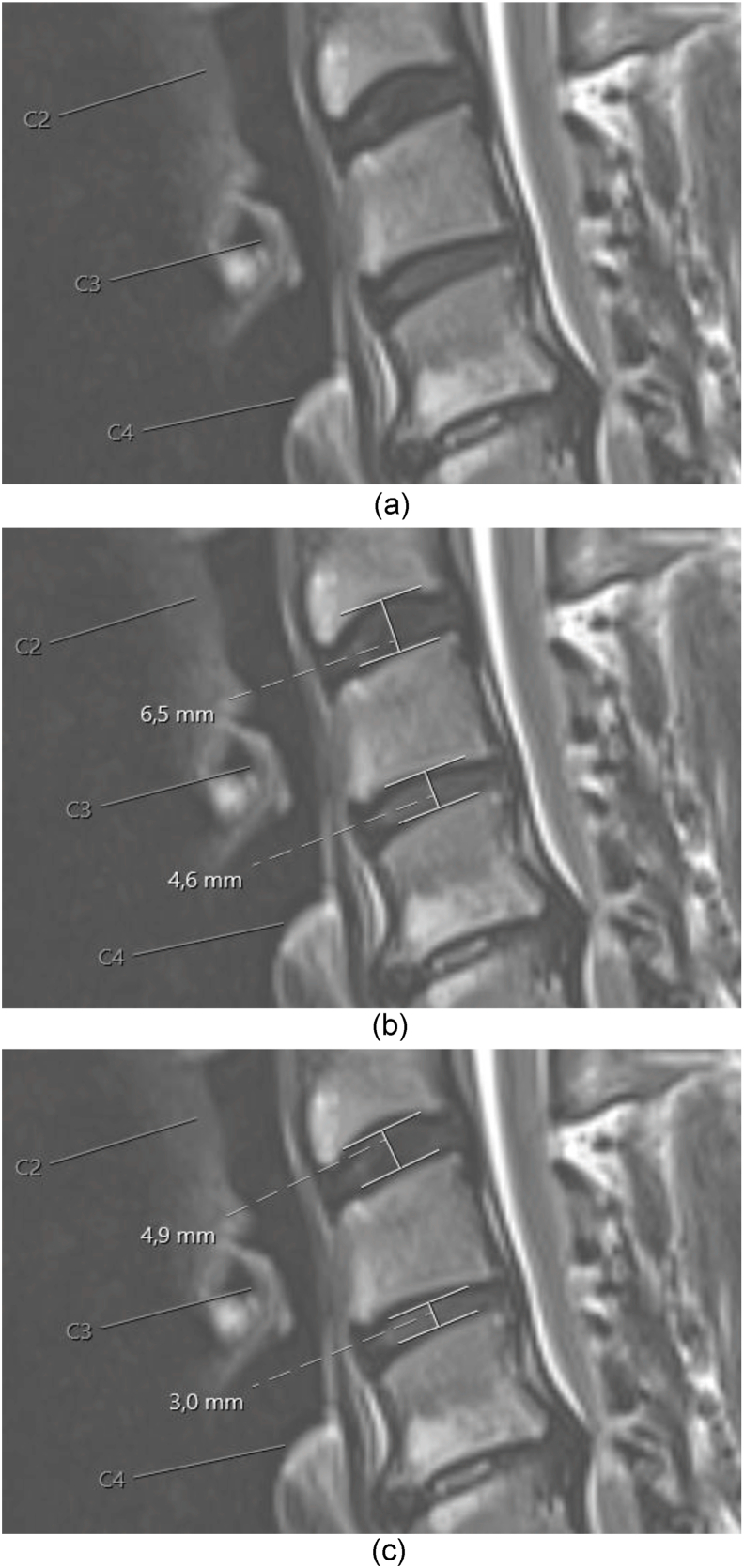
Example of variation in measuring technique on an MRI; (a) MRI without measurements; (b) Mid intervertebral disc measurement from superior outline cancellous bone to inferior outline cancellous bone on level C2-C3 (6.5 mm) and C3-C4 (4.6 mm); (c) Mid intervertebral disc measurement from superior outline nucleus pulposi to inferior outline nucleus pulposi on level C2-C3 (4.9 mm) and C3-C4 (3.0 mm).

The observed discrepancies in disc height measurements between both MRI and X-ray compared with CT are remarkable. Jentsch et al. compared MRI with CT for lumbar intervertebral disc height measurements and reported that MRI measurements were, on average, 1.76 mm higher than those obtained with CT (Jentzsch et al., 2024). They suggest that the discrepancy arises from better imaging contrast, enabling clearer identification of disc and cartilage structures. In line with their suggestion, our findings reveal that the challenge of identifying anatomical structures on MRI results in a systematic bias in disc height estimation, contingent upon the selected landmark. The distinction between cortical bone and cartilaginous endplate is challenging to discern on T2-weighted MRI images, as both structures lack water content and therefore appear hypointense. In cases of disc degeneration, the hypointense region expands, and in severe degeneration, the entire disc may present as hypointense. Figure A4 presents two examples of severe cervical spine degeneration. The nucleus pulposus is more readily identified due to its higher water content, resulting in a hyperintense signal for the less degenerated levels. For the first five patients, disc height measurements on MRI were repeated with an adjusted technique, using the outer line of the nucleus pulposus as landmark rather than the outer line corresponding to cancellous bone, as illustrated in Figures A5b and A5c. On average, the new disc height measurements were 2.3 mm lower than the original measurements. After consultation with a neuroradiologist, we concluded that cortical bone cannot be reliably distinguished from the cartilaginous endplate within the hypointense outline observed on MRI. All MRI derived measurements were thus considered unsuitable for assessing cervical intervertebral disc height.

The observed difference of 1.3 mm between X-ray and CT can be explained by the difference in imaging technique between the two modalities. Conventional X-ray uses diverging cone-beam geometry and is therefore subject to depth-dependent magnification. This magnification error depends on the distance of the X-ray source to the object of interest and image receptor, and increases as the object moves closer to the source. In contrast, CT reconstructs images using geometrically corrected, near-parallel projection, allowing true-scale linear measurements (Goldman, 2007). CT is well established for its accuracy in detecting cervical spine trauma (Holmes and Akkinepalli, 2005). However, its limitations include higher radiation exposure and greater cost compared to X-ray. Ravi et al. found that the anteroposterior vertebral body lengths of C2 and C5 measured on X-rays were, on average, 1.22 mm greater than those measured on CT and MRI (Ravi and Rampersaud, 2008). Similarly, Shigematsu et al. reported a magnification of approximately 18.5 % for C2 and 20.7 % for C5 when comparing X-ray with MRI (Shigematsu et al., 2013). This magnification error for lateral cervical X-rays necessitates cautious interpretation of cervical intervertebral disc height measurements. Moreover, the selection of an imaging modality remains a balance between the advantages and disadvantages of each modality, guided by the clinical setting.

**Fig. A6:**
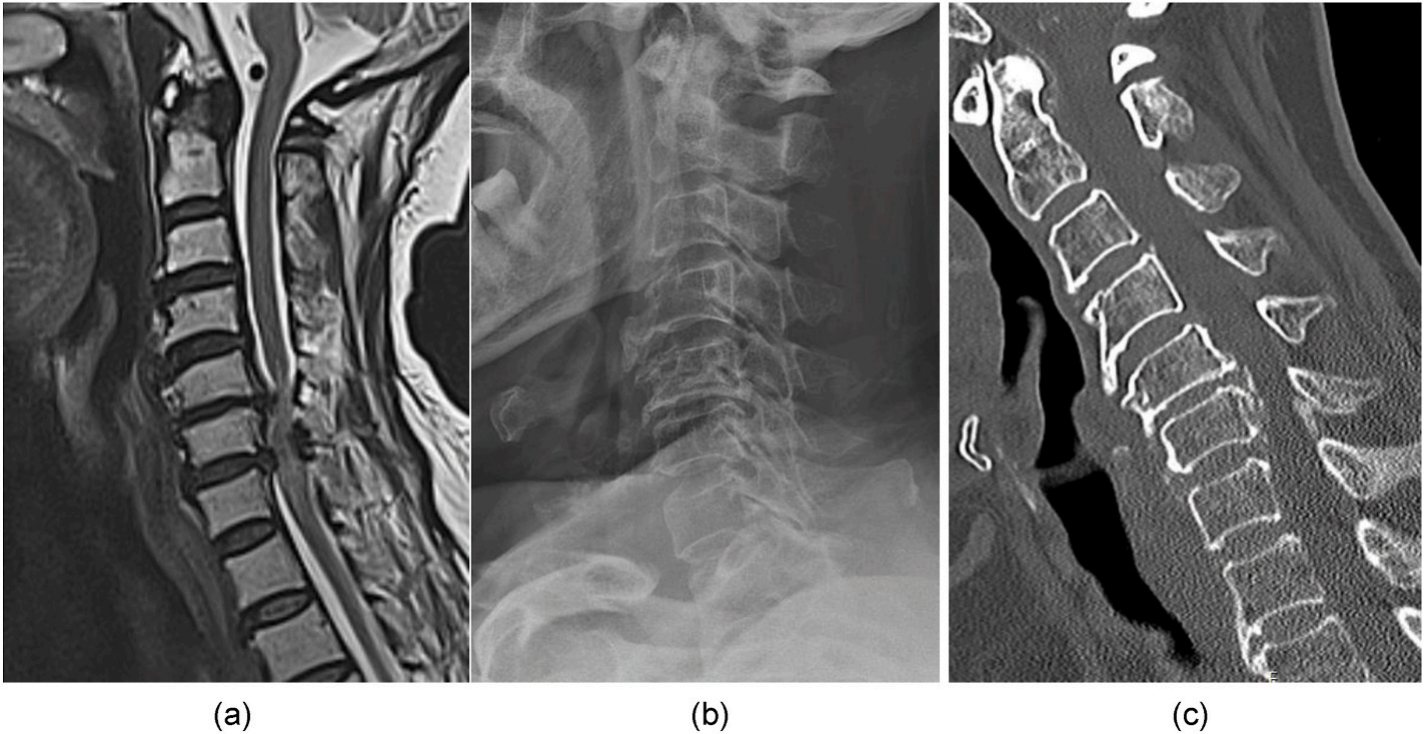
Example of a single patient showing variation in cervical alignment between MRI and X-ray compared to CT. X-ray and CT were made om the same day, 4 days after the MRI was made; (a) MRI; (b) X-ray; (c) CT.

Notably, cervical alignment may differ between MRI, X-ray, and CT. In some cases, alignment appeared lordotic on MRI and X-ray, but kyphotic on CT. Figures A6a, A6b, and A6c illustrate an example of such variation in alignment across modalities in a single patient. This variation can partially be attributed to patient positioning (van Santbrink et al., 2025). X-rays are often acquired in a weight-bearing, standing position, whereas CT and MRI are performed in the supine position, potentially with support of the head. However, this does not explain the difference between MRI and CT. Such a difference in cervical alignment could potentially influence disc height measurements.

A limitation of this study is its retrospective design, which precluded documentation of patient positioning. Additionally, the Kellgren-Lawrence score showed low reliability, likely due its subjective nature and the heterogeneous population with a high degree of degeneration. Nevertheless, this diversity reflects a realistic clinical setting in which intervertebral disc height assessment is commonly performed. The intraobserver reliability was based on 20 participants selected at regular intervals (P5, P10, P15, etc.). This selection method inadvertently included a relatively high proportion of cases affected by trauma or surgical procedures, which resulted in a limited number of intraobserver measurements. Moreover, the single center design of this study limits its generalizability.

## 5 Conclusion

MRI and X-ray exhibit a systematic bias in the measurement of intervertebral disc height when compared to CT. MRI is particularly biased in cases of severe degeneration, and magnification error remains an inherent limitation of X-ray. MRI, X-ray, and CT disc height measurements are reliable. CT provides the most accurate measurements and should be considered when precise disc height measurement is critical. The mid-disc measurement is recommended to ensure high reliability.

## Author contributions

All authors made substantial contributions to this work. All authors were responsible for conceptualization and methodology. E. v.S. and N.K. contributed to data curation and formal analysis. E. v.S. carried out the investigation and drafted the original manuscript. T.B., V.S., R.d.B., A.S., and H. v.S. supervised the study and contributed to critical review and editing of the manuscript. All authors participated in the interpretation of data, revised the manuscript for important intellectual content, and approved the final version for submission.

## Declaration of generative AI and AI-assisted technologies in the writing process

During the preparation of this work the author(s) used ChatGPT in order to improve readability and language. After using this tool, the author(s) reviewed and edited the content as needed and take(s) full responsibility for the content of the publication.

## Funding

This research did not receive any specific grant from funding agencies in the public, commercial, or not-for-profit sectors.

## Conflict of interest

The authors declare the following financial interests/personal relationships which may be considered as potential competing interests: Henk van Santbrink reports a relationship with B Braun Medical Inc that includes: funding grants. If there are other authors, they declare that they have no known competing financial interests or personal relationships that could have appeared to influence the work reported in this paper.

## Figures and Tables

**Table A1:** Description Kellgren-Lawrence score.

1	Doubtful	Minimal anterior osteophytes
2	Minimal	Definite anterior osteophytes; possible narrowing of disc space and some endplate sclerosis
3	Moderate	Moderate narrowing of disc space; definite sclerosis of vertebral endplates and osteophytes
4	Severe	Severe narrowing of disc space; sclerosis of vertebral endplates. multiple large osteophytes

**Table A2:** Inter-class correlation for absolute agreement, Intraobserver reliability without sensitivity analysis for equal slice selection.

ICC intraobserver		MRI (95 %CI)	X-ray (95 %CI)	CT (95 %CI)	n
**C2-C3**	**Mid.** **Ant.** **Post**	.651 (.294–.849) .651 (.284–.851) .370 (−.097 to .701)	.848 (.649–.939) .532 (.139–.786) .583 (.195–.815)	.560 (.142–.806) .882 (.722–.953) .403 (−.007 to .710)	17
**C3-C4**	**Mid.** **Ant.** **Post**	.842 (.626–.938) .792 (.524–.917) .649 (.270–.853)	.929 (.823–.973) .853 (.650–.942) .616 (.223–.837)	.967 (.915–.988) .723 (.402–.886) .800 (.528–.923)	16
**C4-C5**	**Mid.** **Ant.** **Post.**	.693 (.308–.885) .735 (.388–.902) .591 (.160–.838)	.935 (.819–.978) .857 (.635–.949) .754 (.425–.909)	.633 (.215–.858) .410 (−.096 to .751) .711 (.275–.897)	13
**C5-C6**	**Mid.** **Ant.** **Post.**	.405 (−.389 to .832) .385 (−.416 to .825) .387 (−.388 to .823)	.958 (.678–.992) .891 (.394–.977) .879 (.553–.971)	.656 (.022–.911) .563 (−.143 to .883) .816 (.411–.954)	7
**C6-C7**	**Mid.** **Ant.** **Post**	.890 (.581–.974) .831 (.445–.959) .760 (.228–.941)	.932 (.261–.988) .963 (.028–.995) .953 (.811–.989)	.982 (.929–.996) .905 (.593–.978) .910 (.660–.979)	7

Reliability is scored as <.4 = Poor, .4–.6 = Moderate, .6–.8 = Good, >.8 = Excellent.

**Table A3:** Inter-class correlation for absolute agreement, Interobserver reliability without sensitivity analysis for equal slice selection.

ICC interobserver		MRI (95 %CI)	X-ray (95 %CI)	CT (95 %CI)	n
**C2-C3**	**Mid.** **Ant.** **Post.**	.677 (.562–.766) .744 (.616–.828) .613 (.378–.754)	.669 (.486–.783) .792 (.713–.852) .552 (.411–.667)	.836 (.770–.883) .820 (.749–.872) .766 (.678–.833)	114
**C3-C4**	**Mid.** **Ant.** **Post**	.923 (.887–.947) .846 (.780–.893) .677 (.439–.806)	.833 (.588–.917) .842 (.775–.891) .723 (.591–.813)	.875 (.683–.938) .810 (.731–.868) .850 (.785–.896)	101
**C4-C5**	**Mid.** **Ant.** **Post.**	.887 (.833–.924) .763 (.662–.838) .835 (.750–.891)	.754 (.484–.869) .869 (.808–.912) .759 (.656–.835)	.877 (.620–.945) .837 (.762–.890) .801 (.712–.864)	90
**C5-C6**	**Mid.** **Ant.** **Post.**	.864 (.794–.911) .795 (.694–.865) .689 (.486–.809)	.847 (.567–.930) .872 (.805–.916) .623 (.361–.774)	.877 (.793–.925) .831 (.741–.891) .737 (.590–.832)	77
**C6-C7**	**Mid.** **Ant.** **Post**	.925 (.884–.952) .869 (.802–.915) .733 (.571–.833)	.852 (.683–.922) .885 (.825–.925) .690 (.365–.836)	.862 (.672–.931) .891 (.833–.929) .515 (.332–.661)	77

Reliability is scored as <.4 = Poor, .4–.6 = Moderate, .6–.8 = Good, >.8 = Excellent.

**Table A4:** Inter Class Correlation for absolute agreement, Intraobserver reliability before and after sensitivity analysis for equal slice selection.

		CT = (95 %CI)	CT ≠ (95 %CI)	n	MRI = (95 %CI)	MRI ≠ (95 %CI)	n
***C2-C3***	***Mid.*** ***Ant.*** ***Post.***	.898 (.505–.974) .841 (.373–.957) .795 (.432–.936)	.560 (.142–.806) .882 (.722–.953) .403 (−.007 to .710)	11/17	.639 (.247–.855) .695 (.331–.880) .707 (.348–.886)	.651 (.294–.849) .651 (.284–.851) .370 (−.097 to .701)	15/17
***C3-C4***	***Mid.*** ***Ant.*** ***Post***	.980 (.934–.994) .668 (.202–.891) .882 (.593–.967)	.967 (.915–.988) .723 (.402–.886) .800 (.528–.923)	11/16	.949 (.851–.983) .911 (.752–.970) .808 (.516–.933)	.842 (.626–.938) .792 (.524–.917) .649 (.270–.853)	13/16
***C4-C5***	***Mid.*** ***Ant.*** ***Post.***	.983 (.938–.996) .836 (.493–.956) .905 (.591–.977)	.633 (.215–.858) .410 (−.096 to .751) .711 (.275–.897)	9/13	.993 (.875–.999) .934 (.783–.982) .892 (.665–.969)	.693 (.308–.885) .735 (.388–.902) .591 (.160–.838)	10/13
***C5-C6***	***Mid.*** ***Ant.*** ***Post.***	.940 (.727–.989) .861 (.403–.975) .808 (.054–.967)	.656 (.022–.911) .563 (−.143 to .883) .816 (.411–.954)	6/7	.950 (.683–.993) .920 (.531–.988) .891 (.465–.984)	.405 (−.389 to .832) .385 (−.416 to .825) .387 (−.388 to .823)	5/7
***C6-C7***	***Mid.*** ***Ant.*** ***Post***	.982 (.910–.997) .909 (.431–.985) .941 (.726–.989)	.982 (.929–.996) .905 (.593–.978) .910 (.660–.979)	6/7	.890 (.581–.974) .831 (.445–.959) .760 (.228–.941)	.890 (.581–.974) .831 (.445–.959) .760 (.228–.941)	7

= means after sensitivity analysis, ≠ means before sensitivity analysis. Reliability is scored as <.4 = Poor, .4–.6 = Moderate, .6–.8 = Good, >.8 = Excellent.

**Table A5:** Inter Class Correlation for absolute agreement, Interobserver reliability before and after sensitivity analysis for equal slice selection.

		CT = (95 %CI)	CT ≠ (95 %CI)	n	MRI = (95 %CI)	MRI ≠ (95 %CI)	n
***C2-C3***	***Mid.*** ***Ant.*** ***Post.***	.949 (.891–.973) .881 (.818–.922) .908 (.861–.940)	.836 (.770–.883) .820 (.749–.872) .766 (.678–.833)	80/114	.915 (.839–.951) .859 (.668–.929) .791 (.466–.900)	.677 (.562–.766) .744 (.616–.828) .613 (.378–.754)	78/114
***C3-C4***	***Mid.*** ***Ant.*** ***Post***	.914 (.777–.958) .816 (.724–.879) .865 (.794–.912)	.875 (.683–.938) .810 (.731–.868) .850 (.785–.896)	75/101	.960 (.937–.975) .886 (.824–.927) .845 (.520–.933)	.923 (.887–.947) .846 (.780–.893) .677 (.439–.806)	71/101
***C4-C5***	***Mid.*** ***Ant.*** ***Post.***	.928 (.759–.969) .847 (.761–.904) .889 (.823–.931)	.877 (.620–.945) .837 (.762–.890) .801 (.712–.864)	64/90	.905 (.850–.941) .828 (.735–.891) .870 (.774–.923)	.887 (.833–.924) .763 (.662–.838) .835 (.750–.891)	66/90
***C5-C6***	***Mid.*** ***Ant.*** ***Post.***	.933 (.880–.961) .815 (.704–.886) .806 (.512–.909)	.877 (.793–.925) .831 (.741–.891) .737 (.590–.832)	56/77	.919 (.864–.952) .869 (.764–.926) .828 (.636–.912)	.864 (.794–.911) .795 (.694–.865) .689 (.486–.809)	53/77
***C6-C7***	***Mid.*** ***Ant.*** ***Post***	.912 (.714–.963) .907 (.844–.945) .854 (.626–.932)	.862 (.672–.931) .891 (.833–.929) .515 (.332–.661)	53/77	.939 (.898–.964) .897 (.826–.940) .768 (.517–.880)	.925 (.884–.952) .869 (.802–.915) .733 (.571–.833)	54/77

= means after sensitivity analysis, ≠ means before sensitivity analysis. Reliability is scored as <.4 = Poor, .4–.6 = Moderate, .6–.8 = Good, >.8 = Excellent.
